# Editorial: Signaling in stress sensing and resistance in parasitic protozoa

**DOI:** 10.3389/fcimb.2022.962047

**Published:** 2022-07-29

**Authors:** Arijit Bhattacharya, Christopher Fernandez-Prada, Guillermo Daniel Alonso, Arunima Biswas

**Affiliations:** ^1^ Deptartment of Biological Sciences, Adamas University, Kolkata, India; ^2^ Département de Pathologie et Microbiologie, Faculté de Médecine Vétérinaire, Université de Montréal, Saint-Hyacinthe, QC, Canada; ^3^ CONICET Instituto de Investigaciones en Ingeniería Genética y Biología Molecular Dr. Héctor N. Torres (INGEBI), Buenos Aires, Argentina; ^4^ Molecular Cell Biology Laboratory, Deptartment of Zoology, University of Kalyani, Kalyani, India

**Keywords:** trypanosomatids, stress response, drug resistance, signalling, oxidative damage

Protozoan parasites are a large and highly diverse group of unicellular eukaryotes infecting humans and animals, posing enormous health and socio-economic impacts. More than 1 million deaths annually are caused globally by protozoan parasites (Andrews et al., 2014). A significant proportion of the world population is at risk of being afflicted by parasitic diseases like malaria, African trypanosomiasis, Chagas disease, and leishmaniases, widely considered “Neglected global infectious diseases” for which the dearth of clinically approved vaccines and safer and efficacious chemotherapeutic options (https://www.who.int/health-topics/neglected-tropical-diseases#tab=tab_1) still persists. Protozoan pathogens lead complex life cycles, from one host to another, surviving in a variety of morphotypic forms through this complex continuum. The developmental stages can be free-living, extracellular parasitic or intracellular parasitic. The key trigger identified for such transitions has often been stresses such as the alteration of temperature or pH, hypoxia, oxidative burst, or nutrient depletion (Vonlaufen et al., 2008). Of note, stress-associated modulations confer endurance to drug exposure to augment resistance or develop quiescent or ‘persister-like’ forms. Hence, the ability to sense and respond to stress and thrive in less than optimal conditions, hence, are crucial for pathogenicity of the parasites, manifestation of diseases and for combating chemotherapeutic challenge (Bhattacharya et al., 2020). Some of the major stress-response pathways, characterized to date, are elicited by posttranslational modifications (PTM), redox systems, chaperon proteins that mediate protein folding and secondary messengers like cyclic adenosine monophosphate or calcium (cAMP or Ca^2+^) which modulate chemotaxis, antioxidant defence of the parasite (Kelly et al., 2021; Quintana et al., 2021). Against this backdrop, the primary goal of the Research Topic has been to envision the link between adaptive stress response with pathogenicity and drug response. With three original articles, the Research Topic offers a glance hinting towards the significance of PTM in oxidative stress response in *T. cruzi*, alteration of membrane dynamics in drug resistance in *L. donovani*, and development of a potential nanoformulation inducting hallmarks of stress response in *L. donovani*. Alongside this, an illustrative review highlights a putative trypanosmatid G-protein coupled receptor signaling in a sensing host environment.

Chagas disease, endemic in 21 countries across Latin America, registers significantly higher morbimortality compared to other parasitic diseases (Bonney, 2014). The causative agent, *T. cruzi*, experiences a heteroxenous life cycle with a transition between insect vectors and vertebrate hosts. In this course, the parasite encounters variable environmental stress which includes nutritional stress, temperature, and oxidative stress during the infection cycles. Stress sensing and adaptive responses are intricately linked to the differentiation of the parasite into morphological forms like epimastigotes, metacyclic trypomastigotes, or amastigotes. Being an obligatory intracellular parasite, *T. cruzi* must withstand its own metabolic by-products and also cope with the oxidative burst from the host immune system, which includes the production of reactive oxygen species (ROS) and reactive nitrogen intermediates (RNI). Santos Moura et al. describe a novel aspect of posttranscriptional regulation of mitochondrial superoxide dismutase (SODA), a major antioxidant enzyme of the parasite. With an aim to explore the biological significance of their earlier profiling of lysine-acetylated proteins in procyclic and bloodstream forms of *T. cruzi* epimastigotes, where a number of anti-oxidant proteins were identified (Moretti et al., 2018), the group delves with acetylation of the mitochondrial protein TcSODA. Acetylation of K97 is identified to be crucial in modulating SODA activity. The molecular dynamic simulation demonstrated conformational change and altered affinity towards superoxide (O2^•–^) resulting from K97 acetylation. In this context, a mitochondrial lysine deacetylase- TcSir2rp3 was explored for possible involvement in the process. TcSir2rp3 interacts with TcSODA and overexpression of TcSir2rp3 resulted in elevated SOD activity with reduced ROS levels both in mitochondria and cytosol. Using mutated versions of TcSODA and TcSir2rp3 the possible cross talk in the context of K97 acetylation was confirmed, which also affects responsiveness to benznidazole and nifurtimox. The study for the first-time evidenced significance of acetylation in modulating antioxidant enzyme action in Trypanosomatids and reinforced the importance of PTM in modulating stress response.

Stress response has been determined as a preliminary hallmark for the identification of preclinical candidates against pathogens (Egwu et al., 2021). Das et al. here describe the development of a novel nano-formulation using a liposomal curcuminoid HO-3867 which poses cytotoxicity on the extracellular promastigotes and intracellular amastigotes of *L. donovani*. The work is of particular interest in formulating a safe, specific, and effective therapeutic treatment, still an unaccomplished mission against visceral leishmaniasis. HO-3867 was encapsulated with phosphatidylcholine (PC) with stearylamine (SA) by thin film rehydration to prepare PC-SA/HO-3867 liposomes. The formulation induced four-fold upsurge in abrupt ROS production in the parasite coupled to altered lipid metabolism in the promastigotes as reflected by enhanced accumulation of lipid bodies. When tested on *L. donovani* promastigotes, the liposomal preparation induced substantial alteration of morphology and prompted stress induced apoptosis like event typically marked by cell cycle arrest with an increase of sub-G_o_ population, mitochondrial depolarization, and DNA fragmentation. Stress induced activation of metacaspase and poly [ADP]-ribose polymerase-1 (PARP-1) have recently been linked to impaired sirtuin (SIR2) function with depletion of NADH (Purkait et al., 2015). PC-SA/HO-3867challege eventuated 3.5 and 1.6-fold up-regulation of metacaspase and PARP1, respectively. *In vivo* studies in mouse infection model demonstrated RNI and ROS mediated killing of intracellular *L. donovani* amastigotes by PC-SA/HO-3867. Owing to its potential of selectively triggering stress response in the parasite, PC-SA/HO-3867 is a potential candidate for further preclinical analysis.

Albeit alteration of gene copy number and acquisition of mutations have been the cornerstones of drug resistance in *Leishmania*, benchmarking resistance with respect to adaptive response has been attempted for all approved antileishmanials. Chaperone activity, alteration of abundance for glycerophospholipid, lactate, amino acid and amino acid conjugates, or elevated intracellular thiol content have been reported to correlate with the level of resistance as evidenced by comparative transcriptomics, metabolomics, and proteomics data (Gutierrez Guarnizo et al., 2021). Untargeted metabolomics and lipidomic analysis of miltefosine (MIL) and amphotericin B (AmB) resistant lines and isolates revealed significant changes in the levels of sterols, sphingolipids, phospholipids with cyclopropanated fatty acids, and inositol phosphoceramide species (Vincent et al., 2014; Fernandez-Prada et al., 2016; Pountain and Barrett, 2019). In this Research Topic, Ghosh et al. further illuminate the relevance of membrane physicochemical property by impedance spectral analysis. Characterization of 20 independent clinical isolates of *L. donovani* comprising low to high susceptibility to sodium stibogluconate (SSG) for cross-resistance to paromomycin (PMM), AmB or MIL identified six isolates to be cross-resistant to MIL. The association of intracellular thiol content and antimony resistance, as projected earlier (Singh et al., 2014), was initially validated in the isolates with a strong correlation between thiol content and SSG susceptibility. In the quest for unique drug-resistance biomarkers, electrochemical impedance spectroscopy (EIS) has earlier been applied in monitoring their chemoresistivity and sensitivity to drugs (Crowell et al., 2020). Adopting a similar approach, the authors profiled impedance spectra for the isolates with variable susceptibility to MIL and established the correlation between specific impedance spectral patterns with MIL susceptibility. Overall, the SSG-R isolates having a high EC_50_ value against MIL displayed significantly enhanced membrane capacitance owing to higher membrane fluidity. The observation was further reinforced with the *in vivo* MIL responsive patterns in hamster models. The report sheds light on the significance of the systemic biophysical approach in identifying drug responsiveness, however, whether such impedance spectra can be exploited in conjunction with artificial intelligence/machine learning for prompt identification of resistant isolates warrants further introspection.

A number of signaling cascades, in ancestral forms with respect to higher eukaryotic signal transduction pathways, have been associated with stress responsiveness, particularly in sensing the altered environment in the insect vector and in mammalian hosts. A surge of cyclic nucleotide-mediated response has been detected under temperature and pH stress, hypoxia, and depletion of nutrients like purines (Sen Santara et al., 2013; Saha et al., 2020; Kelly et al., 2021). In recent years, flagellar cAMP has surfaced as a major well-defined response in trypanosomatids with identified effectors like cAMP response proteins (CARPs) (Shaw et al., 2022). Though canonical G-protein coupled receptors (GPCR) are apparently absent in trypanosomatids, putative orthologues with reduced domains have been annotated. Diaz et al. offers an illustrative purview on the presence of a GPCR-like signaling system involved in chemotaxis in response to host-derived neuropeptides. Special emphasis is offered to possible components of G-protein coupled signaling like adenylate cyclases, phosphodiesterases, and CARPs. Also, a detailed update on GTPase family proteins from trypanosomatids and noncanonical GPCR like oligopeptide sensing protein-family like TbGPR89, involved in quorum sensing like events (Rojas et al., 2019), have been provided. The review further explores the possibilities of a functional neuropeptide sensing as chemoattractant or chemorepellant *via* RMAP-2/-3 GPCR system in *Leishmania*. Though the hypothesis offered by the authors awaits experimental validation, the review portrays novel dimensions of environmental stress sensing in trypanosomatids.

Advances in the field of stress sensing and signaling would continue to bolster our understanding of the pathogenicity and resistance mechanisms of parasitic protozoa. Systemic identification of stress-associated remodeling would escalate the drug development and management of parasitic protozoan disorders.

## Author contributions

All authors listed have made a substantial, direct, and intellectual contribution to the work and approved it for publication.

## Funding

This study was partially funded by the SERB, Govt. of India (SRG/2020/000702) granted to AB-lab. Work in the CFP-Lab was supported by a Natural Sciences and Engineering Research Council of Canada (NSERC) Discovery Grant RGPIN-2017-04480 and by the Canada Foundation for Innovation (www.innovation.ca), grant numbers 37324 and 38858. Work in the ABi lab was supported by DST-INSPIRE Funding (IFA-12 LSBM-22).

## Acknowledgments

We thank the authors of this special issue for their valuable contributions.

## Conflict of interest

The authors declare that the research was conducted in the absence of any commercial or financial relationships that could be considered as a potential conflict of interest.

## Publisher’s note

All claims expressed in this article are solely those of the authors and do not necessarily represent those of their affiliated organizations, or those of the publisher, the editors and the reviewers. Any product that may be evaluated in this article, or claim that may be made by its manufacturer, is not guaranteed or endorsed by the publisher.
